# Programmed Intermittent Epidural Bolus Reduces Workloads in Labor Analgesia: A Single Center’s Experience

**DOI:** 10.3390/medicina60060993

**Published:** 2024-06-17

**Authors:** Chia-Hung Ou, Wei-Ting Chen

**Affiliations:** Department of Anesthesiology, Shin Kong Memorial Wu Ho-Su Hospital, No. 95, Wen Chang Road, Shih Lin District, Taipei 111, Taiwan; jarhonau@gmail.com

**Keywords:** continuous epidural infusion, epidural analgesia, labor analgesia, patient-controlled epidural analgesia, programmed intermittent epidural bolus, workload

## Abstract

*Background and Objectives:* Labor epidural analgesia can be maintained through programmed intermittent epidural bolus (PIEB), continuous epidural infusion (CEI), or patient-controlled epidural analgesia (PCEA). Our department changed from CEI+PCEA to PIEB+PCEA as the maintenance method. The higher hourly dose setting in the current regimen brought to our concern that side effects would increase with proportional staff workloads. This study aimed to investigate the validity of our proposal that PIEB+PCEA may function as a feasible tool in reducing the amount of work in the obstetrics anesthesia units. *Materials and methods:* This 2-year retrospective review included parturients with vaginal deliveries under epidural analgesia. We compared the staff burden before and after the switch from CEI (6 mL/h, PCEA 6 mL lockout 15 min, group A) to PIEB (8 mL/h, PCEA 8 mL lockout 10 min, group B). The primary outcome was the difference of proportion of parturients requiring unscheduled visits between groups. Side effects and labor and neonatal outcomes were compared. *Results:* Of the 694 parturients analyzed, the proportion of those requiring unscheduled visits were significantly reduced in group B (20.8% vs. 27.7%, chi-square test, *p* = 0.033). The multivariate logistic regression showed that PIEB was associated with fewer unscheduled visits than CEI (OR = 0.53, 95% CI [0.36–0.80], *p* < 0.01). Group B exhibited a significantly lower incidence of asymmetric blockade, as well as motor blockade. In nulliparous subjects, obstetric anal sphincter injury occurred less frequently when PIEB+PCEA was used. Significantly more multiparous women experienced vacuum extraction delivery in group B than in group A, and they had a longer second stage of labor. *Conclusions:* The PIEB+PCEA protocol in our study reduced workloads in labor epidural analgesia as compared to CEI+PCEA, despite that a higher dose of analgesics was administered. Future studies are warranted to investigate the effect of manipulating the PIEB settings on the labor outcomes.

## 1. Introduction

Epidural analgesia represents the gold standard in the management of labor pain, as it is unlikely to be associated with any significant adverse effects for the parturient or fetus [1]. After the indwelling epidural catheter has been sited, epidural solutions, typically a local anesthetic with an opioid, may be administered by means of continuous epidural infusion (CEI), intermittent epidural boluses (IEBs), or patient-controlled epidural analgesia (PCEA).

The addition of CEI onto PCEA diminishes the “peaks and valleys” of analgesia observed in the latter, and it accounts for the enduring popularity of this combination despite the occurrence of undesirable motor blockade, as well as increased need for assisted vaginal delivery [1,2,3]. Programmed intermittent epidural bolus (PIEB), a more recent technique provided by automatic pumps that deliver analgesics at set intervals, has the advantages of reducing breakthrough pain, decreasing risks of motor blockade, lowering the need for instrumental vaginal delivery, and improving maternal satisfaction without interfering laboring and neonatal outcomes, possibly through its ability to improve the spread of analgesics within the noncontiguous epidural space [4,5,6,7,8].

In our institution, we changed from CEI+PCEA to PIEB+PCEA in August 2022, after reviewing recent high-quality studies comparing the two modes. However, the first-line acute pain service (APS) staff kept raising concerns about increased workloads (e.g., resiting epidural catheter or requirement of rescue analgesia). Following the approval of our quality-assurance committee, we adjusted the PIEB+PCEA regimen two months after. Nonetheless, the higher hourly dose setting in the current regimen, compared to the previous CEI+PCEA configurations, brought to our concern that side effects would increase with proportional staff workloads. This led us to conduct a before–after retrospective study to investigate the validity of our proposal that PIEB may function as a feasible tool in reducing the amount of work in the obstetrics anesthesia units.

## 2. Materials and Methods

### 2.1. Data Sources

After the approval from Shin Kong Memorial Wu Ho-Su Hospital Institutional Review Board (protocol number 20221202R, date of approval: 9 March 2023), we conducted a retrospective review of the electronic medical records for fetal deliveries with **painless labor**, i.e., labor epidural analgesia, before and after the change from CEI+PCEA to PIEB+PCEA. We included all parturients that had vaginal delivery, who were of ASA physical status II, and who were of age 18 or older. Exclusion criteria were deviation of initial pump settings from our standard protocol, fetal delivery within one hour after combined spinal–epidural (CSE) placement, occurrence of unintentional dural puncture, pregnancy term less than 36 weeks, inappropriate PCEA use (triggered by accident or by someone other than the parturient), and missing data. The CSE method consisted of an intrathecal underdosing of local anesthetics, followed by an epidural injection. Deliveries within one hour after CSE were excluded to minimize the effect of subarachnoid anesthetics, considering its potential to confound the outcomes of this study. According to our experience and a previous study [9], the duration of satisfactory labor analgesia with intrathecal bupivacaine was approximately 60 min, and thus the cut-off interval of one hour was determined. Dural puncture epidural technique was not a part of our routine practice, and its effect under the regimen applied in our institution had not been verified before. Hence, no inadvertent dural puncture was included.

In August 2022, the standard maintenance technique was switched from CEI+PCEA to PIEB+PCEA. During the first 2 months, the PIEB regimen was 6 mL/h with unchanged PCEA dosage. We adjusted the regimen to 8 mL/h and, at the same time, PCEA to 8 mL per bolus (lockout 10 min), in October 2022. Data were retrieved from August 2021 to July 2022 and from November 2022 to October 2023. The 3-month interval (from August to October 2022) was determined as a familiarization period, as well as to avoid additional vigilance following the adoption of a new practice.

### 2.2. Study Group and Outcome Measurement

Upon admission to the labor and delivery unit, each female for planned vaginal delivery was provided with a thorough introduction to our painless labor service by the APS personnel. Should she choose to receive painless labor, informed consent was obtained before any intervention was given. Every parturient was then put in lateral decubitus position, and a multi-orifice catheter was inserted through an 18-gauge Tuohy needle (Portex Epidural Minipack SYSTEM 1, ICU Medical, Dublin, OH, USA) up to 6–7 cm within the epidural space by competent senior residents or attending physicians. A test dose of 2 mL 1% lidocaine was administered to rule out intravascular or intrathecal injection. The formula of epidural injectate was ropivacaine 1.6 mg/mL (0.16%) plus fentanyl 2 μg/mL. Painless labor was initiated with an epidural loading dose of 10 mL, with or without a preceding CSE of intrathecal bupivacaine 2 mg in normal saline 2 mL through a 27-gauge Quincke spinal needle (Becton Dickinson, Madrid, Spain). Maintenance was a background CEI (6 mL/h, designated group A) with PCEA 6 mL (lockout 15 min) or PIEB (8 mL/h, designated group B) with PCEA 8 mL (lockout 10 min), using the CADD^®^ Solis Ambulatory Infusion Pump (ICU Medical, Dublin, OH, USA). PIEB began 1 h after the loading dose, while CEI started right after. All boluses were delivered at a fixed rate of 175 mL/h. As for the one-hour maximum dosage, 30 mL injectate was set in CEI and 40 mL in PIEB. For self-administration of PCEA boluses, the safety of the lockout mechanism was carefully explained to the parturients. They were advised to notify the APS staff immediately if pain was not satisfactorily managed with PCEA or if any intolerable side effects occurred.

The APS staff, consisting of one trained nurse anesthetist rotating three shifts over 24 h, would visit each parturient every 2 h until fetal delivery. The function of the staff was to serve as the first responder to all complaints (unscheduled visits) associated with painless labor. They would execute orders from the attending anesthesiologist (additional intervention) and complete the painless labor log for every individual. The numerical verbal pain scores (VPS, 0 = no pain and 10 = worst pain ever), presence of side effects, and all managements provided were documented.

Rescue top-ups were given either through the *“clinician bolus”* function of the pump in 5 mL increments or manually with 5–10 mL 1% lidocaine every time. In case the regimen of the given mode was not sufficient to meet the analgesic requirement of the parturient or causing intolerable side effects, titration or tapering of the maintenance rate would be performed in 1–2 mL/h increments or decrements, respectively. For example, in the event of frequent vomiting, CEI would be adjusted to 4 mL/h or even halted. To counter asymmetric epidural block, catheter withdrawal would be carried out in anticipation to prevent segmented pain relief [10]. Resiting of the catheter would be the last resort in the attempt to overcome suboptimal blockade (e.g., persistent unilateral blockade or patchy block). 

Demographic and obstetric data were collected. The duration of epidural use was calculated from the beginning of loading dose to vaginal delivery of the fetus. The primary outcome was the number of parturients requiring unscheduled visits, as well as the total number of unscheduled visits per subject. A subgroup analysis regarding the parturients receiving additional interventions was performed. Secondary outcomes were the inclusion of incidence of breakthrough pain (labor pain uncontrolled by PCEA that demanded any intervention), side effects, local anesthetic consumption, and obstetric outcomes (as shown by a subgroup analysis).

### 2.3. Statistical Analysis

Sample size was determined by available data. Data were shown as mean (standard deviation, SD), median [interquartile range, IQR], or number (percentage). Outcomes were compared using the *t*-test for normally distributed variables (Welch’s *t*-test if unequal variances), and Mann–Whitney test for non-parametric data. Categorical data were analyzed with chi-squared test or Fisher’s exact test. Odds ratios (ORs) with 95% confidence intervals (95% CI) were calculated as appropriate. A multivariable test with Logistic regression was performed to detect confounders regarding the primary outcome. A *p* < 0.05 was considered statistically significant. A data analysis was performed by SPSS, version 25 (IBM^®^, Armonk, NY, USA).

## 3. Results

### 3.1. Demographics of the Participants

**Figure 1:**
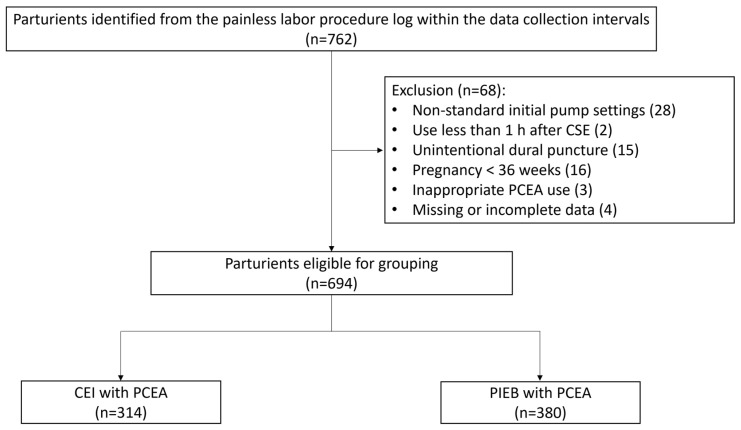
Flowchart of parturient selection. CEI, continuous epidural infusion; CSE, combined spinal–epidural analgesia; PCEA, patient-controlled epidural analgesia; PIEB, programmed intermittent epidural bolus.

We recruited a total of 762 parturients, and 694 of them completed the retrospective study (Figure 1). The demographics and obstetric characteristics are shown in Table 1. There were more parturients in spontaneous labor before painless labor in group B (*p* = 0.01). Group A had less baseline cervical dilation (1.74 vs. 1.91, centimeter, *p* = 0.03), gestational age of the fetus (38.14 vs. 38.44, week, *p* < 0.01), and neonate birth weight (2972 vs. 3030, gram, *p* = 0.02). The work proportion of each attending obstetrician caring for parturients was similar in both groups.

### 3.2. Analysis for Unscheduled Visits and Additional Interventions

For primary outcome (Table 2), the proportion of parturients requiring unscheduled visits was significantly reduced in group B (20.8% vs. 27.7%, *p* = 0.033; OR = 0.69, 95% CI [0.48–0.97]). The multivariate logistic regression showed a significant predictive value of maintenance with PIEB for the probability of requiring unscheduled visits (OR = 0.53, 95% CI [0.36–0.80], *p* < 0.01) after adjusting for all variables of interest (Table 3). The APS staff performed a total of 323 additional interventions on the studied population (Table 4). In the PIEB subgroup, the frequency of tapering the painless labor dose was significantly higher, and that of catheter withdrawal was lower (*p* < 0.01).

### 3.3. Comparisons of Secondary Outcomes

For breakthrough pain, the difference in rate did not show statistical significance between group A and B (14.6% vs. 10%, *p* = 0.06; Table 2). Group B exhibited a significantly lower incidence of asymmetric blockade (16.8% vs. 28%, *p* < 0.01), as well as motor blockade (7.1% vs. 12.1%, *p* = 0.03). The consumption of the epidural solution was significantly higher in group B.

With respect to obstetric outcomes (Table 5), subjects were segmented according to parity. The rate of episiotomy was higher with PIEB regardless of parity. In nulliparous subjects, obstetric anal sphincter injury (OASIS) occurred less frequently under PIEB (30.8% vs. 53.4%, *p* < 0.01). Significantly more multiparous women experienced vacuum-extraction delivery (VED) in PIEB (25.5% vs. 14.6%, *p* = 0.03), and they had a longer second stage of labor (28 vs. 19 min, *p* < 0.01).

## 4. Discussion

In our study, we found that PIEB substituting for CEI resulted in a 6.9% reduction in women requiring unscheduled visits, as well as the pain service workload in labor analgesia. Furthermore, the overall number of extra visits per parturient, whether time-weighted, also differed significantly. This would suggest a consistent beneficial impact of PIEB on work capacity. We believed our work was the first to study and compare the effect of PIEB+PCEA at a higher dosage with CEI+PCEA in the influence of workloads.

With regard to the demographic data, both groups were comparable, although there were some slight disparities. A lesser proportion of women under CEI was noted to have had spontaneous labor before painless labor ensued. Such a finding could be attributed to the data-collection period for group A, as it partly overlapped with a recurrent COVID-19 outbreak in Taiwan [11], during which artificial induction of labor was favored by the obstetric colleagues. It was a part of our obstetric routine to administer induction agents immediately following the initiation of painless labor for those admitted for labor induction. Despite the significant yet small difference in baseline cervical dilation between both groups, most parturients requested epidural analgesia at a relatively early stage of labor (mean dilation was less than 2 cm). These could likely obviate the delay in labor progression for those without spontaneous labor when comparing the two groups. The fetal gestational age and neonate birth weight were both increased in group B, but the mean differences were again too small to be considered clinically significant.

Another effort to avert the possible confounding effect of the discrepancy noted in the demographics between the two groups was the multivariate logistic regression for the primary outcome. As shown in Table 3, both the extent of pre-epidural cervical dilation and artificial induction of labor, although different among the two groups, were not associated with unscheduled visits. In addition to PIEB, CSE independently lowered the odds for parturients to ask for additional intervention, most likely due to its rapidity of onset, which improved the quality of the labor analgesia [9]. Lastly, higher consumption of epidural analgesics was also related to more unscheduled visits, probably as a result of rescue boluses during events of breakthrough pain that required the attendance of APS personnels.

Few studies directly investigated the effect of PIEB on healthcare workloads. Rinaldi and colleagues studied the anesthetist work time (minutes spent on catheter placement and patient management) as an indicator of personnel burden when implementing PIEB in labor analgesia [12]. However, the control group received manual intermittent bolus as maintenance. In a recent retrospective study by Tsao et al. comparing PIEB to CEI, they reported an increase in mean extra visits and a 1.6-time more manual boluses and dose adjustment of the pump [13]. However, the ambiguity of their dosing strategy could hint a lack of a well-designed study protocol. The PCEA lockout intervals were also much longer (30 & 15 min), which could result in inadequate analgesia during active phase of labor. We believed these factors would cause bias and lead to controversial extrapolation of their findings. Comparatively, we had included in detail all additional interventions and endeavor to provide a more comprehensive analysis on work amount. Although we found a higher proportion of tapering the maintenance dose in group B, it could be the result of the minor increase, yet insignificant, of pruritus, nausea or vomiting in the secondary outcomes. On the other hand, there was more withdrawal of the catheter in group A. We practiced the technique of leaving up to 6–7 cm of the catheter within the epidural space, which was about 1.5 times the length of others [5,6,14]. It was reported deeper insertion would prevent premature dislodgement [15]. The incidence of asymmetric blockade in epidural anesthesia varied widely among different groups (5.4~30.2%) [4,16,17], and it was likely influenced by inter-study heterogeneity in research design, definition, or technique of maintaining analgesia. There was evidence of positive correlation between the insertion depth of a multi-orifice catheter and segmented blockade [17]. The overall incidence in our study (152 in 694, 21.9%) could be attributed to our practice of catheter insertion. D’Angelo et al. reported manipulation of the catheter was effective in decreasing lateralization [10]. However, such practice would unequivocally result in an increase in work amount. It is our institutional culture that we routinely perform catheter withdrawal to ensure the blockade was consummate. Further prospective studies are required to verify the efficacy of such practice.

We reported a 10% of breakthrough pain in the parturients receiving PIEB, which was at the lower end of the spectrum in previous studies (12~34.9%) [4,7,14]. In our secondary outcomes, there was a trend of fewer breakthrough pain with PIEB although it was not significant. This could arise from underreport of breakthrough pain during the active phase of first stage and the second stage of labor, especially in those with a rapid delivery. Also, in our study, the multiparous parturients under CEI had a significantly shorter second stage of labor.

The side effects in group B were in consistency with existing evidence [8], despite the higher hourly dose and PCEA bolus dose. Ginosar et al. reported bolus administration of epidural fentanyl resulted in reduced systemic uptake [18], and our findings were in favor of their work. In our study, the slight increase in the epidural solution used (0.8 mL/h) in group B was likely the result of the dose reduction effect of this mode shown in previous studies [8], as the discrepancy on hourly consumption should be at least 2 mL/h according to the default setting (CEI, 6 mL/h; PIEB, 8 mL/h).

PCEA lockout intervals are generally considered mandatory for concerns of safety. Unsuccessful PCEA attempts during lockouts were used as a surrogate of unmet analgesia [14]. In our study, the PCEA dose used and the unsuccessful attempts were similar in both groups. It appeared difficult to interpret these results, as the lockout interval was less in group B. Further delineation of the above findings was necessary before a consensus could be attained.

Our findings on obstetric outcomes required interpretation with caution. In Taiwan, there was an extremely high incidence of episiotomy (100%) at term delivery [19]. It was around 80% in our institution. The reported rate of VED in all live births in Taiwan was 10.81% in 2020 [20]. Our inhouse obstetricians had a strong tendency to perform VED (up to 40% in this study), and this would likely lead to a high incidence of OASIS [21]. Zuo and colleagues found that, with incremental doses of PIEB, there appeared to be a step-up in the rate of episiotomy and duration of the second stage of labor [14]. They speculated that the women under higher doses might not have the urge to push, and the resultant prolonged second stage of labor could increase the incidence of episiotomy. This could probably be the situation in our findings. Following the introduction of our PIEB regimen, the obstetrician in charge might tend to lower the standard of threshold to perform episiotomy and VED in the anticipation of exhausted parturients and longer second stage of labor of the parturient. Zuo et al. included only nulliparous women, and epidural analgesia was discontinued during the second stage of labor in more than half of the subjects. Our studied population comprised parturients with mixed parity, and the data on the discontinuation of painless labor during the second stage were unavailable. In a large retrospective analysis by Munro et al., PIEB caused an increase in operative deliveries for parturients with mixed parity compared with CEI [22]. Their epidural regimens were not very different from ours. More studies powered to investigate the obstetric outcomes of the multiparous in PIEB are needed. Also, nulliparity had been identified as a risk factor for OASIS [21], and our results supported this finding. We also noted that PIEB seemed to exert a protective effect against OASIS. It was likely the higher incidence of severe perineal lacerations among nulliparous women that highlighted this effect, which was not reproduced in the multiparous subgroup. The mechanism behind this phenomenon remained elusive.

The retrospective and single-centered nature of our study posed certain limitations, and the interpretation of the results should be within the context of this research. While randomized controlled trials (RCTs) had been long considered superior to observational research in comparative studies, their designs could affect the generalizability of the results [23]. RCTs comparing PIEB and CEI often included nulliparous women only [6,14], subjecting the studies to participation bias. Study protocols usually resulted in an increased attentiveness by the staff (Hawthorne effect), and this might impose a challenge to reflect actual workloads in a real-world setting. Moreover, it is difficult to carry out a blind study for this topic because of the distinctive interaction between PIEB and PCEA (a lockout was triggered by each programmed bolus), which was not observed in CEI+PCEA. Therefore, we believed that an observational study was more appropriate. 

## 5. Conclusions

Our PIEB+PCEA protocol reduced staff workloads in epidural labor analgesia as compared to CEI+PCEA despite the fact that a higher dose of analgesics was administered. Further modifications to our regimen may be necessary to fully realize its potentials. Future studies are warranted in the clarification of the effect of manipulating the PIEB settings on the labor outcomes.

## Figures and Tables

**Table 1:** Parturient characteristics.

	CEI (*n* = 314)	PIEB (*n* = 380)	*p*-Value
Age (year)	33.1 (4.6)	32.8 (4.9)	0.43
Body mass index (kg/m^2^)	26 (3.4)	26.5 (3.7)	0.07
Parity	0 [0–1]	0 [0–1]	0.61
Indication of admission to the labor and delivery unit			0.01
Induction of labor	225 (71.7%)	236 (62.1%)	
Spontaneous labor	89 (28.3%)	144 (37.9%)	
Baseline cervical dilatation (cm)	1.8 (1)	1.9 (1)	0.03
Spontaneous rupture of membrane before painless labor	39 (12.4%)	42 (11.1%)	0.58
Gestational age (week)	38.1 (1)	38.4 (1)	<0.01
Neonate birth weight (gram)	2972 (316)	3030 (335)	0.02
Baseline VPS	2 (2.6)	2.1 (2.9)	0.53
CSE	14 (4.5%)	13 (3.4%)	0.48
Duration of epidural analgesia used (min)	721 (575)	727 (565)	0.89
Attending obstetrician			0.13
No. 1	25 (8%)	28 (7.4%)	
No. 2	31 (9.9%)	43 (11.3%)	
No. 3	162 (51.8%)	169 (44.5%)	
No. 4	43 (13.7%)	46 (12.1%)	
No. 5	33 (10.5%)	52 (13.7%)	
No. 6	19 (6.1%)	42 (11%)	

Data are mean (SD), median [IQR], or n (percentage). CEI, continuous epidural infusion; CSE, combined spinal–epidural; PIEB, programmed intermittent epidural bolus; VPS, verbal pain score.

**Table 2:** Analgesia outcomes and side effects.

	CEI (*n* = 314)	PIEB (*n* = 380)	*p*-Value
Extra visits (number of subjects)	87 (27.7%)	79 (20.8%)	0.033
Extra visits (number per subject)	0 [0–1]	0 [0–0]	0.036
Extra visits (number per subject per day)	0 [0–0.99]	0 [0–0]	0.038
Consumption of epidural solution			
Total (mL)	109 (81)	124 (89)	0.02
Time-weighted (mL/h)	10.6 (5.2)	11.4 (3.5)	0.02
Manual bolus dose (mL)	0.8 (3)	0.7 (3.5)	0.73
“*Clinician bolus*” dose (mL)	0.3 (1.7)	0.1 (1.1)	0.13
Breakthrough pain	46 (14.6%)	38 (10%)	0.06
Side effects			
Asymmetric blockade	88 (28%)	64 (16.8%)	<0.01
Motor blockade (Bromage score > 0)	38 (12.1%)	27 (7.1%)	0.03
Nausea or vomiting	21 (6.7%)	39 (10.3%)	0.10
Pruritus	14 (4.5%)	29 (7.6%)	0.08
Urinary retention	21 (6.7%)	19 (5%)	0.34
High level block (above T6 level)	6 (1.9%)	3 (0.8%)	0.34
Hypotension (SBP < 20% of baseline)	3 (1%)	3 (0.8%)	1.00

Data are mean (SD), median [IQR], or n (percentage). CEI, continuous epidural infusion; PCEA, patient-controlled epidural analgesia; PIEB, programmed intermittent epidural bolus; SBP, systolic blood pressure.

**Table 3:** Multivariate logistic regression: odds of requiring unscheduled visits.

Variables	OR	95% CI	*p*-Value
PIEB mode	0.53	0.36–0.80	<0.01
Total consumption of the epidural solution (mL)	1.01	1.01–1.02	<0.01
Duration of analgesia (min)	1.00	0.99–1.00	0.66
CSE	0.25	0.10–0.64	<0.01
Baseline cervical dilation (cm)	0.84	0.66–1.07	0.16
Baseline VPS	0.96	0.87–1.05	0.33
Induction of labor	0.71	0.44–1.15	0.17
Spontaneous rupture of membrane before painless labor	0.78	0.42–1.45	0.44
Duration of 2nd stage of labor (min)	0.99	0.99–1.00	0.51

CSE, combined spinal–epidural; 95% CI, 95% confidence interval; OR, odds ratio; PIEB, programmed intermittent epidural bolus; VPS, verbal pain score.

**Table 4:** Subgroup analysis on additional interventions.

Cumulative Additional Interventions Count	CEI (*n* = 169)	PIEB (*n* = 154)	*p*-Value
Titrating dose	31 (18.3%)	22 (14.3%)	0.33
Tapering dose	22 (13%)	41 (26.6%)	<0.01
Manual bolus	31 (18.3%)	31 (20.1%)	0.68
*“Clinician bolus”* function of the pump	8 (4.7%)	6 (3.9%)	0.71
Resiting of the catheter	11 (6.5%)	10 (6.5%)	0.99
Pausing the pump	3 (1.8%)	3 (1.9%)	0.91
Withdrawal of the catheter	53 (31.4%)	28 (18.2%)	<0.01
Medications for side effects	10 (6%)	13 (8.5%)	0.38

Data are n (percentage). CEI, continuous epidural infusion; PCEA, patient-controlled epidural analgesia; PIEB, programmed intermittent epidural bolus.

**Table 5:** Obstetric outcomes.

	Nulliparous			Multiparous		
	CEI (*n* = 191)	PIEB (*n* = 227)	*p*-Value	CEI (*n* = 123)	PIEB (*n* = 153)	*p*-Value
Maternal						
Duration of 2nd stage of labor (min)	60 (43)	65 (49)	0.30	19 (13)	28 (26)	<0.01
Mode of delivery			0.10			0.03
Spontaneous delivery	97 (50.8%)	97 (42.7%)		105 (85.4%)	114 (74.5%)	
VED	94 (49.2%)	130 (57.3%)		18 (14.6%)	39 (25.5%)	
Episiotomy	159 (83.2%)	210 (92.5%)	<0.01	75 (61%)	116 (75.8%)	<0.01
Degree of perineal laceration	3 [2–3]	2 [2–3]	<0.01	2 [2–2]	2 [2–2]	0.67
OASIS	102 (53.4%)	70 (30.8%)	<0.01	13 (10.6%)	17 (11.1%)	0.89
Postpartum hemorrhage	4 (2.1%)	4 (1.8%)	1.00	2 (1.6%)	4 (2.6%)	0.70
Fetal						
Apgar score, 1 min	8 [8–9]	8 [8–9]	0.82	9 [8–9]	9 [8–9]	0.36
Apgar score, 5 min	9 [8–9]	9 [8–9]	0.30	9 [9–9]	9 [9–9]	0.49

Data are mean (SD), median [IQR], or n (percentage). CEI: continuous epidural infusion; OASIS, obstetric anal sphincter injury; PIEB, programmed intermittent epidural bolus; VED, vacuum extraction delivery.

## Data Availability

The data presented in this study are available upon request from the corresponding author. (The data are not publicly available due to the policy of the National Health Insurance Administration in Taiwan.)

## References

[1] Callahan E.C., Lee W., Aleshi P., George R.B. (2023). Modern labor epidural analgesia: Implications for labor outcomes and maternal-fetal health. Am. J. Obstet. Gynecol..

[2] Heesen M., Böhmer J., Klöhr S., Hofmann T., Rossaint R., Straube S. (2015). The effect of adding a background infusion to patient-controlled epidural labor analgesia on labor, maternal, and neonatal outcomes: A systematic review and meta-analysis. Anesth. Analg..

[3] van der Vyver M., Halpern S., Joseph G. (2002). Patient-controlled epidural analgesia versus continuous infusion for labour analgesia: A meta-analysis. Br. J. Anaesth..

[4] McKenzie C.P., Cobb B., Riley E.T., Carvalho B. (2016). Programmed intermittent epidural boluses for maintenance of labor analgesia: An impact study. Int. J. Obstet. Anesth..

[5] Wong C.A., Ratliff J.T., Sullivan J.T., Scavone B.M., Toledo P., McCarthy R.J. (2006). A randomized comparison of programmed intermittent epidural bolus with continuous epidural infusion for labor analgesia. Anesth. Analg..

[6] Capogna G., Camorcia M., Stirparo S., Farcomeni A. (2011). Programmed intermittent epidural bolus versus continuous epidural infusion for labor analgesia: The effects on maternal motor function and labor outcome. A randomized double-blind study in nulliparous women. Anesth. Analg..

[7] Fidkowski C.W., Shah S., Alsaden M.R. (2019). Programmed intermittent epidural bolus as compared to continuous epidural infusion for the maintenance of labor analgesia: A prospective randomized single-blinded controlled trial. Korean J. Anesthesiol..

[8] Hussain N., Lagnese C.M., Hayes B., Kumar N., Weaver T.E., Essandoh M.K., Reno J., Small R.H., Abdallah F.W. (2020). Comparative analgesic efficacy and safety of intermittent local anaesthetic epidural bolus for labor: A systematic review and meta-analysis. Br. J. Anaesth..

[9] Okutomi T., Saito M., Mochizuki J., Kuczkowski K.M. (2009). Combined spinal-epidural analgesia for labor pain: Best timing of epidural infusion following spinal dose. Arch. Gynecol. Obstet..

[10] D’Angelo R., Berkebile B.L., Gerancher J.C. (1996). Prospective examination of epidural catheter insertion. Anesthesiology.

[11] Centers for Disease Control, Taiwan (2022). Statistics for Severe Pneumonia with Novel Pathogens (COVID-19), Nationwide, Indigenous and Imported. https://nidss.cdc.gov.tw/nndss/disease?id=19CoV.

[12] Rinaldi L., Ghirardini A.M., Troglio R., Bellini V., Donno L., Biondini S., Biagioni E., Baciarello M., Bignami E., Girardis M. (2021). Pain management during labor: Use of intermittent drug delivery devices for improvement of obstetric and neonatal outcome and reduction of healthcare burden: A large non-inferiority randomized clinical trial. J. Anesth. Analg. Crit. Care.

[13] Tsao S.-L., Li W.-T., Chang L.-Y., Yeh P.-H., Yeh L.-T., Liu L.-J., Yeh C.-B. (2023). Assessing Continuous Epidural Infusion and Programmed Intermittent Epidural Bolus for Their Effectiveness in Providing Labor Analgesia: A Mono-Centric Retrospective Comparative Study. Medicina.

[14] Zuo R.H., Dang J.J., Zhuang J.W., Chen Q.M., Zhang J.Y., Zheng H.W., Wang Z. (2022). The incidence of breakthrough pain associated with programmed intermittent bolus volumes for labor epidural analgesia: A randomized controlled trial. Int. J. Obstet. Anesth..

[15] Königsrainer I., Bredanger S., Drewel-Frohnmeyer R., Vonthein R., Krueger W.A., Königsrainer A., Unertl K.E., Schroeder T.H. (2009). Audit of motor weakness and premature catheter dislodgement after epidural analgesia in major abdominal surgery. Anaesthesia.

[16] Ferrer L.E., Romero D.J., Vásquez O.I., Matute E.C., Van de Velde M. (2017). Effect of programmed intermittent epidural boluses and continuous epidural infusion on labor analgesia and obstetric outcomes: A randomized controlled trial. Arch. Gynecol. Obstet..

[17] Souvatzis X., Diamantaki E., Korda D., Dermitzakis E., Zaganas I., Tzanakis N.E. (2013). Predictors of laterality of motor block during epidural analgesia in a mixed surgical population. Acta Anaesthesiol. Scand..

[18] Ginosar Y., Riley E.T., Angst M.S. (2003). The site of action of epidural fentanyl in humans: The difference between infusion and bolus administration. Anesth. Analg..

[19] Graham I.D., Carroli G., Davies C., Medves J.M. (2005). Episiotomy rates around the world: An update. Birth.

[20] Health Promotion Administration, Ministry of Health and Welfare, Taiwan (2021). 2020 Statistics of Birth Reporting System. https://www.hpa.gov.tw/File/Attach/14674/File_17440.pdf.

[21] Hsieh W.C., Liang C.C., Wu D., Chang S.D., Chueh H.Y., Chao A.S. (2014). Prevalence and contributing factors of severe perineal damage following episiotomy-assisted vaginal delivery. Taiwan J. Obstet. Gynecol..

[22] Munro A., MacCormick H., Aidemouni M., Nash C.M., George R.B. (2022). A retrospective cohort comparison of programmed intermittent epidural bolus (PIEB) and continued epidural infusion (CEI) on delivery mode. Can. J. Anaesth..

[23] Black N. (1996). Why we need observational studies to evaluate the effectiveness of health care. BMJ.

